# DNA Damage Repair Pathways in Prostate Cancer: A Narrative Review of Molecular Mechanisms, Emerging Biomarkers and Therapeutic Targets in Precision Oncology

**DOI:** 10.3390/ijms241411418

**Published:** 2023-07-13

**Authors:** Ioanna-Maria Grypari, Vasiliki Tzelepi, Kostis Gyftopoulos

**Affiliations:** 1 Cytology Department, Aretaieion University Hospital, National Kapodistrian University of Athens, 11528 Athens, Greece; jogrypari@aretaieio.uoa.gr; 2 Department of Pathology, School of Medicine, University of Patras, 26504 Patras, Greece; btzelepi@upatras.gr; 3 Department of Anatomy, School of Medicine, University of Patras, 26504 Patras, Greece

**Keywords:** prostate cancer, DNA damage repair, homologous recombination deficiency, mismatch repair pathway, biomarkers

## Abstract

Prostate cancer (PCa) has a distinct molecular signature, including characteristic chromosomal translocations, gene deletions and defective DNA damage repair mechanisms. One crucial pathway involved is homologous recombination deficiency (HRD) and it is found in almost 20% of metastatic castrate-resistant PCa (mCRPC). Inherited/germline mutations are associated with a hereditary predisposition to early PCa development and aggressive behavior. BRCA2, ATM and CHECK2 are the most frequently HRD-mutated genes. BRCA2-mutated tumors have unfavorable clinical and pathological characteristics, such as intraductal carcinoma. PARP inhibitors, due to the induction of synthetic lethality, have been therapeutically approved for mCRPC with HRD alterations. Mutations are detected in metastatic tissue, while a liquid biopsy is utilized during follow-up, recognizing acquired resistance mechanisms. The mismatch repair (MMR) pathway is another DNA repair mechanism implicated in carcinogenesis, although only 5% of metastatic PCa is affected. It is associated with aggressive disease. PD-1 inhibitors have been used in MMR-deficient tumors; thus, the MMR status should be tested in all metastatic PCa cases. A surrogate marker of defective DNA repair mechanisms is the tumor mutational burden. PDL-1 expression and intratumoral lymphocytes have ambivalent predictive value. Few experimental molecules have been so far proposed as potential biomarkers. Future research may further elucidate the role of DNA damage pathways in PCa, revealing new therapeutic targets and predictive biomarkers.

## 1. Introduction

Prostate cancer (PCa) has been largely known as an androgen-dependent malignancy since 1942, when Hodges and Huggins first recognized the strong connection between androgens and PCa progression [1], and it is now well established that its pathogenesis and progression are mediated by the androgen receptor (AR) [2]. A particularity of PCa is that it usually carries only a small amount of point mutations and is more frequently characterized by chromatin remodeling alterations through chromosomal translocations and gene deletions, in contrast to other neoplasms (melanoma, lung cancer, etc.) that develop after exposure to specific chemical carcinogens [3,4,5]. The revealing of this peculiar molecular signature has created the term “chromoplexy” in order to depict the complexity of the chromosomal rearrangements and copy number alterations seen in these neoplasms [4,6]. Metastatic PCa carries more copy number alterations and mutations in general, compared with localized disease, indicating the heterogeneity of PCa in different progression stages [5,7]. 

The most common genetic abnormality, present in 40–50% of prostate cancers [8], is the translocation of the transmembrane protease serine 2 to the v-ets erythroblastosis virus E26 oncogene (TMPRSS2-ETS) [9,10]. The result of this fusion is the overexpression of a fragmented form of the ERG protein, which belongs to the ETS transcriptional family, resulting in the subsequent activation of gene transcription, which causes DNA damage [7,11]. The second most common genetic alteration in PCa is speckle-type pox virus and zinc finger protein (SPOP) mutation, which is mutually exclusive to TMPRSS2-ERG translocation and, among other actions, is responsible for the activation of the PI3K/mTOR cascade [9]. The latter leads to transcriptional events similar to the ones observed in BRCA1 mutations, causing dysfunction of the homologous recombination (HR) mechanisms responsible for double-strand DNA repair [12]. 

RNA sequencing derived from PCa cell lines and gene co-expression network analysis through bioinformatics revealed a positive correlation between 17 DNA repair genes and androgen treatment, supporting the notion that the androgen receptor can regulate the expression of DNA repair genes and justifying the resistance to radiation in a proportion of PCa cases [13]. However, there is no universal agreement in the literature concerning the genes that participate in this interaction with AR [13].

Overall, DNA damage is an important and complicated mechanism and the consequences of its contribution to carcinogenesis are not fully clarified yet. In this review article, we summarize the up-to-date evidence on the role of DNA repair pathways in PCa, underlying the significance of currently used tissue biomarkers and their optimal detection methods. Our aim is to highlight novel therapeutic targets and potential predictive and prognostic biomarkers along these pathways, which represent valuable tools for precision oncology. 

## 2. Homologous Recombination Deficiency

Homologous recombination deficiency (HRD) is a term describing tumor phenotypes in which the ability to repair DNA double-strand breaks utilizing the homologous recombination repair (HRR) pathway is lost [14]. Lately, precision medicine has been focusing on targetable mutations, although their frequency in tumors may be very low. The most notable mutations that can be targeted in PCa include gene products that regulate DNA repair through homologous recombination (HR), such as BRCA1, BRCA2, ATM, PALB2, CHEK2 and HOXB13 [9,15,16,17]. 

HRR is part of the DNA damage repair (DDR) pathway that also includes base excision repair, nucleotide excision repair, mismatch repair and non-homologous end joining [18,19]. HRR is activated during the S and G2 phases of the cell cycle and is a very efficient and error-free process, in contrast to non-homologous end joining, which repairs errors at any phase of the cell cycle and has a propensity for errors [18]. The importance of such mechanisms is substantial, as it defines the fate of the cell after DNA damage [20]. After exposure to carcinogens, including endogenous or exogenous factors such as ultraviolet and ionizing radiation and chemical pollutants that cause oxidative stress, DDR is activated before the cell commences replication [5,20]. DDR recruits tumor suppressor proteins and restrains the aggregation of genomic alterations [19]. If the damage is extensive, then cell death occurs, since the accumulation of double- and single-strand breaks may lead to genomic instability, which carries a risk of malignant transformation [5,20,21]. 

DNA repair enzymes are found mutated in a variable proportion of PCa cases, ranging from 5–10% in localized disease to almost 20% in advanced, castrate-resistant or metastatic disease [18,19,20,22,23,24,25,26]. Analysis of 333 cases of primary PCa retrieved from The Cancer Genome Atlas (TCGA) demonstrated that mutations in HR genes were detected in 19% of the samples, being present even at the early stages of the disease [5,27]. The most common alterations in the TCGA database were ATM mutations (4%), RAD51C deletions (3%), BRCA2 deletions or mutations (3%), CKD12 deletions or mutations (2%), BRCA1 mutations (1%) and FANCD2 aberrations (6%) [5]. In a study including 150 patients with recurrent or metastatic PCa, without any further selection, 14% of the patients were found to harbor pathogenic mutations for enzymes that mediated DNA damage repair in their tumors and BRCA2 was the most commonly affected gene [26,28]. HRD gene aberrations are considered an early-stage event in PCa, but their more frequent occurrence in advanced disease is explained by their association with a poor prognosis [29] and their role in disease progression [19]. 

### 2.1. Germline vs. Sporadic Mutations in HR Genes

Alterations in HR genes are most commonly sporadic but can also be germline [30]. The term *inherited PCa* refers to families that fulfill the John Hopkins criteria, these being the following: (a) at least three first-degree relatives diagnosed with PCa, (b) the presence of the disease in three consecutive generations and (c) early-onset disease in two family members [31]. 

It is well established that a family history of PCa increases the risk for all male relatives, even in the absence of characteristic genetic alterations [32,33]. These cases, comprising 15–20% of all patients, are best described with the term “family-associated PCa” and should not be confused with hereditary PCa, which occurs less frequently, at an estimated 5% [33], and includes a population with a specific molecular profile—for instance, *BRCA1/2* or *HOXB13* mutation carriers [32]. A proposed theory is that the predisposition in families with a higher incidence of PCa probably occurs due to the interplay between common polymorphisms of intermediate and low penetrance in various genes with environmental risk factors that enhance inflammation [33]. 

A hereditary predisposition to PCa should be suspected when there is a family member diagnosed with PCa at an age < 60 years or with an aggressive disease course, a family history of more than three malignancies related to hereditary breast/ovarian cancer or Lynch syndrome or, finally, in men of Ashkenazi Jewish origin [34] and if two distinct histological patterns are seen in a prostate biopsy: intraductal carcinoma of the prostate (IDCp) and cribriform histology (see below) [28,32,35,36].

The PRACTICAL study analyzed germline mutations in men predisposed to PCa and demonstrated that mutations that are considered pathogenic or likely pathogenic in genome databases (for instance, ClinVar) were linked with a worse prognosis, a fact that was not observed for mutations classified as variants of uncertain significance. This means that the detection of a mutation or variant alone is not informative, and further characterization of the genetic alteration detected is required in order to obtain accurate predictive information for the patient [37]. 

Germline mutations in DDR have distinct behavior and malignant potential compared with sporadic cases [5]. Somatic mutations can develop after progression to metastatic CRPC (mCRPC) [24], while germline mutations are inherited through an autosomal dominant pathway with incomplete penetrance [18]. Germline *BRCA2* mutations have especially been associated with a poor prognosis and have been found to be an independent prognostic factor for PCa patients [38,39], and this applies even in cases with a limited tumor volume and low histopathological grade [24]. These neoplasms demonstrate higher genomic instability and more copy number alterations [24], including *MYC* amplification, which is known to correlate with aggressive behavior and rapid disease progression [40]. The aggressiveness of *BRCA2*-mutated neoplasms has been attributed to the fact that these tumors develop a subpopulation of cells that are castrate-resistant and can grow independently, even after the administration of antiandrogen therapy. This model is supported by research data that show a similar molecular signature in *BRCA2*-mutated tumors and metastatic CRPC, which is only rarely found in sporadic PCa [24]. Another theory is that these neoplasms, due to the DNA repair defects that they harbor, gradually accumulate genetic alterations, in contrast to sporadic cases with functionable DNA repair systems, where DNA defects are properly and timely repaired [24]. It has been proposed that genetic alterations, characterized as truncal, arise at the early stages of carcinogenesis and are carried by all the daughter cells, while later-acquired alterations are present only in specific cell subpopulations, contributing to the heterogeneity and complexity of cancer genetics [32]. 

The presence of germline mutations has additional implications for the relatives of the patient, as they should be tested as potential carriers [22,23,32]. Of interest, 5.5% of men with a familial predisposition to PCa share the same mutational pattern in DNA repair enzymes, such as *BRCA1*, *BRCA2* and *ATM* alterations, even if they have not developed PCa [22]. Experts suggest to start screening for PCa in men with known *BRCA2* and *BRCA1* mutations at the age of 40 [23], supported by studies that have revealed a diagnosis of PCa at as early as 41 years of age in *BRCA2*- and 43 in *BRCA1*-mutated patients [41]. 

### 2.2. BRCA and Non-BRCA Mutations

*BRCA* mutations represent the most common DNA repair alterations in PCa [18] and, among them, the majority of cases show *BRCA2* alterations (12% *BRCA2* alterations versus 2% *BRCA1* in advanced PCa) [5,19,29,32]. The most common *BRCA2* alteration results in the production of a truncated form of the protein, followed by complete deletion of the gene; only a minority of cases show point mutations [32]. In contrast, the most frequent *BRCA1* mutations lead to a truncated gene product and are often accompanied by *TP53* mutations [42].

*HOXB13* was the first gene that was shown to enhance the prostate cancer risk by up to 10 times and was linked with familial cases of PCa and early-onset disease in some [23,31,33] but not all studies [43]. Specifically, the mutant HOXB13G84E has been associated with lower-risk tumor characteristics [43], early-onset disease [33] and European origin among patients [31,33]. Other alterations have been encountered in different populations, such as G135E in Chinese men and variants A128D and F240L in Portuguese men [31]. 

A recent study conducting genome analysis on non-BRCA-mutated PCa showed that germline *ATM* and *CHEK2* alterations had lower penetrance than *BRCA2*. In addition, prostate cancer carried different genetic alterations in these genes compared to breast and ovarian cancer [43]. Germline *CHEK2* mutations increase the risk for PCa development at a moderate level [23] and have been linked with aggressive or high-risk cancer [43]. Genome analysis in patients with non-*BRCA*-mutated familial PCa has confirmed that mutated *ATM* is found in cases with advanced disease, higher PSA levels at the time of initial diagnosis and a high D’Amico score [43]. Further studies need to be performed, mainly for *ATM* and *PALB2*, as the existing data for these two genes in PCa are limited. It is promising, though, that *ATM* aberration augments the sensitivity to PARP inhibitors [44].

CDK12 is a cyclin-dependent kinase that regulates transcription elongation through the phosphorylation of RNA polymerase II, subsequently modifying gene expression and influencing DDR gene expression [45]. *CDK12* is mutated in a small percentage of metastatic castrate-resistant PCa (CRPC), varying from 4.7% to 7%, and, when mutated, it is not combined with HR deficiency and *ATM* or MMR gene mutations [46,47]. Thus, *CDK12*-mutant PCa comprises a distinct molecular group of PCa.

### 2.3. Clinical and Histologic Characteristics of HRD Tumors

*BRCA2*-mutated tumors tend to develop in younger patients, are usually classified as an intermediate or high risk of recurrence, metastasize earlier and are associated with shorter survival, even when treated with prostatectomy or radiotherapy [19,23,24,27,36,41,48,49]. In addition, HRD-targeted therapies have been developed (see below). Thus, tumor testing for HR genes is recommended in all metastatic PCa patients and can be considered in patients with regional disease, especially those with adverse characteristics [50].

The IMPACT study focused on screening men having *BRCA1* and *BRCA2* mutations for the diagnosis of PCa and revealed that the value of prostate-specific antigen (PSA) levels higher than 3.0 ng/mL and of prostate biopsy was greater in the *BRCA2*-mutated population than in the *BRCA2* wild type [41]. In contrast to these data, there are increasing data available showing that PCa with low PSA values at the time of diagnosis is associated with DDR mutations [28], and, in comparison, metastatic cases with a known *BRCA2* [51] but not *BRCA1* [41] mutation present with lower PSA levels compared to their wild-type controls. Another study that tried to elucidate the pathological characteristics of *BRCA2*-mutated tumors exhibited no statistically significant difference compared to *BRCA2* wild-type tumors regarding the TNM classification, prognostic grade group or histology subtype of the tumors [51]. The mutated subgroup, however, had a higher mutational load and recurring *ATM* and *BRCA1* alterations [51]. 

Intraductal carcinoma, which is associated with high-grade and high-stage PCa; the presence of lymph node and distant metastases; and shorter disease-specific and overall survival is more frequently seen in cases of hereditary PCa and often harbors *BRCA1/2* mutations [36,52,53,54,55,56]. Additionally, *BRCA2* mutation carriers have a higher probability of showing IDCp in their biopsy [15,16,24,36,52,57,58]. Even IDCp associated with low-grade PCa has been shown to harbor aberrations in DDR genes, such as *BRCA2*, *CHEK2* and *CDK12*, which are not present in the invasive component [59]. Furthermore, according to recent data, in PCa without IDCp, HRD (estimated by a higher HRD score—see below) results from mutations in DDR genes, in contrast to PCa with IDCp, where HRD is attributed to *TP53* mutations [60]. Whole-genome sequencing of *BRCA2*-mutated and IDCp-harboring PCa revealed the molecular resemblance of these tumors to metastatic CRPC, even at the initial stages of tumorigenesis, and the activation of crucial signaling pathways, such as WNT/b-catenin modulator MED12L/MED12, which have been associated with an adverse prognosis [10,36]. It should be mentioned that these alterations are not found in sporadic cancers with IDCp, while MED12 is absent in normal prostate and organ-confined PCa [36]. 

Apart from intraductal carcinoma, the somatic loss of both alleles of the *BRCA2* gene and increased genomic instability and copy number alterations have also been associated with the cribriform pattern and the ductal type of adenocarcinoma [10,35,58,61]. 

Based on these data, current guidelines suggest that patients with intermediate-risk PCa and IDCp or cribriform histology can be considered for germline or somatic genetic testing for DDR alterations [62,63,64,65,66,67,68,69].

### 2.4. Clinical and Therapeutic Implications of HRD

Based on the aggressiveness of HRD-mutated neoplasms, an earlier and more aggressive therapeutic approach should be followed for *BRCA2*-mutated tumors. Moreover, patients with this molecular signature demonstrate a significant response to platinum-based chemotherapy and poly-adenosine diphosphate (ADP) ribose polymerase (PARP) inhibitors (PARPi) (see below). Regarding platinum-based chemotherapy, HRD has been associated with an increased likelihood of a PSA response in a small cohort (N = 64) of patients with PCa, although no difference in overall survival was seen [70]. However, the number of patients enrolled in this cohort was limited, so these observations need to be validated in larger groups of patients [70,71]. Similarly, a small prospective cohort study showed that patients with germline mutations in DDR experienced better outcomes when treated with abiraterone or enzalutamide, compared to taxanes [38]. In addition, radical prostatectomy, rather than radiotherapy, should be the treatment of choice for these patients in the localized setting [24]. Interestingly, these worrisome features are not detected in *BRCA1* carriers, indicating that the clinical implications of these two mutations are significantly different [41]. 

Patients with CRPC and HRD alterations show promising response rates after treatment with PARP inhibitors [44]. Therefore, in 2016, PARP inhibitors received approval by the Food and Drug Administration (FDA) and were incorporated into the therapeutic schemes of metastatic CRPC [44]. To date, two PARP inhibitors (Olaparib and Rucaparib) have been approved for metastatic CRPC [72,73]. Clinical data support their efficacy, as documented by PSA and circulating tumor cell responses and improved progression-free survival and overall survival [73]. 

One of the first clinical trials that elucidated their utility was the TOPAPR-A trial [74]. The patient group included fifty (50) patients harboring alterations in DNA repair enzymes, previously treated with docetaxel or second-generation androgen deprivation therapy (ADT); treatment with Olaparib resulted in a favorable clinical response [74]. Based on the subsequent PROfound clinical trial (NCT02987543) [75] (a randomized phase 3 clinical trial in 245 patients with a mutation in at least one of *BRCA1*, *BRCA2* and *ATM* and 152 patients with alterations in other HRD genes), Olaparib was approved for patients whose tumors harbor a genetic alteration in *BRCA1*, *BRCA2*, *ATM*, *BRIP1*, *BARD1*, *CDK12*, *CHEK1*, *CHEK2*, *FANCL*, *PALB2*, *PPP2R2A*, *RAD51B*, *RAD51C*, *RAD51D* or *RAD54L* as a second-line therapy after the failure of second-generation antiandrogen agents or docetaxel or as a third-line therapy [23]. Based on the Triton 2 (NCT02952534) and Triton 3 (NCT02975934) clinical trials [76,77], Rucaparib has been approved for tumors harboring *BRCA1/2* mutations, either somatic or germline [78,79]. Currently, ongoing clinical trials are tested the efficiency of other members of the PARP inhibitor family, such as niraparib (clinical trial number: NCT02854436) [23]. 

Proper risk assessment of patients at the time of the initial diagnosis should incorporate the HRD status [60]. Interestingly, different alterations in the genes have recently been found to result in different response rates to treatment [6]. For instance, a PCa patient with a base substitution (c.4211C > G) in *BRCA2* showed a response to radiotherapy and androgen deprivation therapy (ADT) in a Chinese cohort study [80], while patients with CDK12 mutations did not respond well to hormonal therapy, PARPi or taxanes but showed positive (and occasionally durable) responses to PD-1 inhibition [81,82]. On the contrary, ATM and CDK12 mutations do not seem to respond to PARP inhibitors as effectively as BRCA1/2 mutations [83,84]. Despite the fact that this observation was noticed in a small group of patients (46 patients) with progressive metastatic CRPC, and the retrospective nature of the study, it is in accordance with the results of the Triton 3 trial [83]. A possible reason for this difference is that biallelic loss and germline mutations, which are usually detected in BRCA1/2 carriers, respond better to the treatment [83]. This underlines the importance of accurate sequencing in HR genes in order to be fully utilized as both prognostic and predictive biomarkers.

### 2.5. Predictive Biomarkers to PARPi Response

Next-generation sequencing (NGS) seems to be the most appropriate tool to detect alterations in the HRD-associated genes mentioned above. This method detects multiple genetic alterations, including mutations and chromosomal alterations in a single test, although none of the currently available tests is validated to detect germline mutations. Genomic analysis with a high reading depth can raise the awareness of hereditary PCa, and these cases should be referred to genetic counseling that provides an holistic approach and guides the patients through specialized genetic tests [32]. NGS testing can be performed on metastatic tissue or on plasma circulating free DNA (cfDNA) [32]. Patients with mutations in genes other than BRCA1/2, such as *ATM*, *PALB2*, *CHEK2*, *FANCA* and *HDAC2*, are also responding well to PARP inhibitors, underlying the importance of using a broader detection panel [44]. 

A scoring system, called the Homologous Recombination Deficiency Score, has also been established, incorporating several chromosomal aberrations, such as the loss of heterozygosity, telomeric allelic imbalance and large-scale transitions [60]. This score gives, however, a general expression of the HRD status and does not directly reflect which particular enzyme is damaged. Nonetheless, it appears that it can be successfully utilized as a predictive biomarker for the potential response to PARP inhibitors [60]. The presence of *MYC* and *TP53* alterations is also frequently associated with high HRD scores, even without synchronous aberrations in the HR system [60]. The *MYC* oncogene supervises the repair of double-strand DNA breaks [60]. Subsequently, the concurrent inhibition of the MYC pathway along with PARP inhibitors could be beneficial [60].

Regarding the follow-up of patients under PARP inhibitor treatment, a relatively new but promising approach is the whole-exome sequencing of liquid biopsy specimens, which reduces the need for additional surgical interventions in patients [27,71,85,86]. Testing of metastatic tissue poses some practical difficulties, as metastatic foci in PCa are mostly found in the bones, which sometimes are difficult to access; even when the sample is adequate, the DNA that is extracted from this tissue has questionable quality, due to the decalcification that is performed during tissue processing [87]. 

One of the first applications of liquid biopsy in PCa research was conducted in the TOPAPR-A clinical trial, where it was depicted that cfDNA analysis can provide adequate information regarding acquired genetic alterations, even before signs of clinical progression are evident, allowing the early detection of resistance [19]. The broad utility of this approach may lead to modifications of the therapeutic scheme and the discontinuation of non-responsive drugs, avoiding unnecessary toxicity [85]. cfDNA is derived from the circulated tumor DNA and DNA fragments that are produced after cellular death or apoptosis [86]. The main disadvantage of this revolutionary method is the small number of circulating tumor cells in some cases, and, thus, the practical difficulty to isolate and further process them in order to extract DNA [71]. Therefore, liquid biopsy is preferably utilized in advanced PCa cases and not in the early stages of the disease.

### 2.6. PARPi Mechanism of Action 

**Figure 1:**
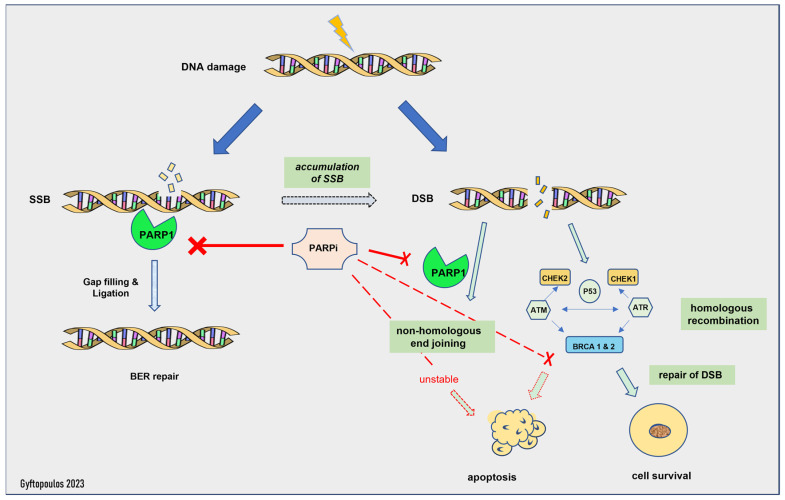
Mechanism of action of PARP inhibitors. SSB: single-strand break, DSB: double-strand break, BER: base excision repair.

The mechanism of action of PARP inhibitors in HRD tumors has been clarified during the last decade [88]. Normally, the PARP complex consists of 16 enzymes and their common feature is the production of poly(ADP-ribose) from NAD, a chemical reaction that generates nicotinamide [88]. Some members of the PARP family, PARP1 and -2, activate the repair mechanisms after DNA damage [72]. In particular, PARP1 can restore double- and single-strand breaks in the nucleotide chain [72], preserving the integrity of the replication fork and, subsequently, of transcription, thus shielding the genome against replication stress [88]. If this process fails, then replication is interrupted, and deadly breaks, followed by cellular death, are induced [88]. Homologous recombination undertakes the correction of double-strand breaks by enlisting various repair enzymes, including BRCA1 and BRAC2 [88]. PARP1 has a central role in the recruitment of other family members, as the absence of PARP1 downgrades the efficacy of PARP inhibitors in general [88] (Figure 1). Furthermore, in experimental models, PARP1 enhances the oncogenic actions of TMPRSS-ERG, through the enrichment of AR-mediated transcription, which eventually drives the cells into a castrate-resistant phase [19]. PARP1 is essential for the activation of ERG [19].

The activity of PARP inhibitors is described as PARP trapping, as it traps PARP1/2 near the region of DNA damage, resulting in the stalling of replication forks. Stalled replication forks lead to highly cytotoxic double-strand breaks that, in HR-proficient cells, are repaired by HR [20,73]. HRD cells are unable to repair the accumulating double-strand breaks and die [22]. Thus, PARP1i is effective only in HRD cells, as the HR-proficient cells can escape its action. This is called the synthetic lethality hypothesis [89]. Synthetic lethality describes a situation where a combination of two events leads to cell death, but each event is individually viable. Olaparib inhibits PARP1 and -2, while Rucaparib is a less selective PARP inhibitor with a broader range of action, including non-PARP targets [88]. Of note, patients with germline mutations in the *BRCA* genes suffer from severe adverse effects and especially myelotoxicity [20], whereas most patients with sporadic HRD face milder toxicity, such as anemia and fatigue [74].

Therapeutic synthetic lethality can also be applied to tumors that have a molecular signature similar to *BRCA*-mutated tumors, even in the absence of homologous recombination deficiency, introducing a new term of “BRCAness”. For example, PARP inhibitors have been shown to induce replication stress in experimental models that exhibit the concurrent loss of *P53* and *RB1* and *MYC* amplification [18,88]. This could explain the favorable response to these drugs, even in the absence of *BRCA* mutations [44]. However, despite this experimental evidence, the MAGNITUDE trial failed to show a survival benefit in men who did not harbor HRR mutations and were treated with PARPi (niraparib) combined with abiraterone [84]. On the other hand, platinum-based chemotherapy, such as docetaxel and cabazitaxel, acts through DNA alkylation, producing DNA strand breaks, thus contributing to synthetic lethality. Therefore, they are widely used in advanced PCa [32], although they do not directly target a specific DNA repair mechanism [19]. 

### 2.7. Biomarkers Predicting PARPi Resistance

Unfortunately, neoplastic cells eventually develop resistance mechanisms that overcome the external PARP inhibition and block the pathway of synthetic lethality, as the PARP enzymes become functionable again [18]. The time frame in which resistance develops is usually after 10–18 months of treatment [19]. It usually happens through the mutational reversion of *BRCA1/2*, most frequently due to single-nucleotide alterations that provoke frame shift modifications and result in HR proficiency, preventing the deaths of neoplastic cells [18]. Alternatively, they protect the replication fork to preserve transcription. Other possible resistance mechanisms include the acquisition of genetic alterations in PARP enzymes or the development of efflux pumps that reduce the concentrations of PARP inhibitors within the cancer cells [18]. In a published case report, acquired resistance due to AKT mutation appeared a few months after Olaparib administration and was handled with a concurrent AKT inhibitor [90].

Recent research work proposes that the *MMS22L* gene (which encodes the DNA repair methyl methanesulfonate-sensitivity 22-like protein) is frequently deleted in PCa, mediates HRR and has predictive value regarding PARP inhibitors’ effectiveness. The suggested mechanism involves the blockage of the RAD51 molecule, an essential moderator of HRR, in a TP53-dependent way [73]. In contrast, the loss of *CHEK2* has been found to increase the resistance to PARP inhibitors, due to the upregulation of BRCA2, and the concomitant use of PARP and ATR inhibitors could overcome this resistance pathway [73]. 

## 3. The Mismatch Repair System and Microsatellite Instability

### 3.1. The Mismatch Repair Pathway

The mismatch repair pathway (MMR) is a highly conserved system that aims to correct spontaneous base–base mispairs and small insertions–deletions (indels) that occur during DNA replication, thus securing the integrity of this process [91,92] and preserving DNA homeostasis and genomic stability [93,94]. MMR is composed of eight genes, *hMSH2*, *hMSH3*, *hMSH5*, *hMSH6*, *hMLH1*, *hPMS1* (*hMLH2*), *hMLH3* and *hPMS2* (*hMLH4*), that work in heterodimers [91]. hMSH2–MSH6 and hMSH2–MSH3 recognize and attach to mismatched bases in the DNA sequence and attract the hMLH1–hPMS2 complex [94,95]. Other molecules are also recruited, e.g., the proliferating cell nuclear antigen and the replication factor C, and the endonuclease activity of PMS2 is activated. The mismatched sequence is excised, followed by the re-synthesis of the correct sequence [92,96]. The hMSH2–MSH6 complex has the ability to identify single base mismatches and dinucleotide indels, while hMSH2–MSH3 has a broader spectrum and can detect indels of up to 13 nucleotides [97]. 

The MMR system is frequently deregulated in cancer (MMR deficiency—dMMR), through the mutation of one of its genes (more commonly MSH2, MSH6, MLH1 and PMS2) or by the hypermethylation-induced silencing of the MLH1 gene [94]. Germline mutations of one of the abovementioned genes or the epithelial cell adhesion molecule (EpCAM) gene (which is located adjacent to the MSH2 gene) are the causes of Lynch syndrome, a hereditary cancer predisposition syndrome characterized by an increased risk of developing various tumors, mainly colorectal carcinoma but also endometrial, gastric, ovarian, pancreatic, urothelial (upper urinary tract), biliary tract and small intestinal carcinomas [98]. Lynch syndrome is also associated with a moderate risk for the development of prostate cancer [23,99] and patients with Lynch syndrome have a two-fold lifetime risk of developing PCa [100]. In sporadic tumors, dMMR is mainly caused by hypermethylation of the MLH1 promoter, resulting in gene silencing and protein loss [101,102]. dMMR, either sporadic or germline, is common in some tumors, such as colorectal and endometrial carcinomas [101,102], but can also be seen, albeit with significantly reduced frequency (approximately 3–22%, depending on the study) [103], in other tumors, including prostate carcinoma [104].

**Figure 2:**
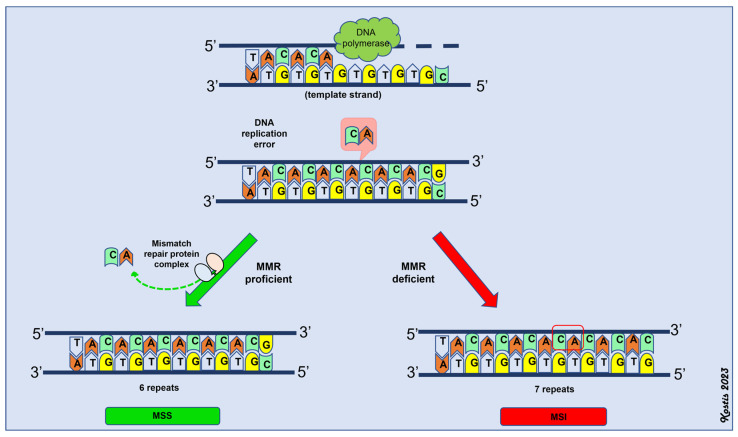
Schematic representation of mismatch repair (MMR) system. MSS: microsatellite stability, MSI: microsatellite instability (MSI-high).

A deficient MMR system, due to mutations or epigenetic modifications, results in a propensity for multiple point mutations across the genome (hypermutability), thereby inactivating tumor suppressor genes and driving tumor initiation and progression [91]. Tumors that develop through the dMMR pathway have a high mutational burden and are also characterized by alterations in the lengths of microsatellites; hence, this pathway is termed microsatellite instability [96]. Microsatellites are repetitive sequences consisting of one to eight nucleotides and are more commonly located near the coding regions of various genes [95,96]. DNA polymerase slippage during DNA replication causes alterations in their lengths through the insertion or deletion of base pairs [105,106,107]. The MMR system is responsible for correcting these types of errors. Thus, the functionality of the MMR system defines the microsatellite status (MS) of the tumor. If the MMR system is working properly (MMR-proficient or pMMR), the tumor is microsatellite stable (MSS) or it has low microsatellite instability (MSI-low) [95]. If there is a deficiency in the MMR system (dMMR), then the tumor (MSI-high) has high microsatellite instability (Figure 2).

The best-studied malignancy that is correlated with dMMR is colon cancer. MSI-high colorectal carcinomas have a better prognosis compared to MSI-low neoplasms [92], and adjuvant treatment may be spared for a subset of patients with MSI-high stage II colorectal carcinomas [108,109]. In addition, MSI-high tumors do not respond well to therapy with 5-FU, oxaliplatin and irinotecan; thus, if adjuvant therapy is needed, other regimens should be used. Lastly, the MSI status of the tumor can predict the patient response to immune checkpoint inhibitors [105,110]. The KEYNOTE-177 study enrolled 307 patients with metastatic MSI-high or dMMR colorectal carcinoma and showed a significant survival advantage for patients that received pembrolizumab compared to the control group that was treated with conventional chemotherapy [111]. Based on these results, the KEYNOTE-158 trial enlisted 233 patients with MSI-high non-colorectal malignancies, such as endometrial, ovarian, urothelial and prostate carcinoma [112], and treated them with an anti-PD-1 agent (pembrolizumab), and the outcome was beneficial, with a decreased tumor burden and a sustainable clinical response in the treatment arm compared to the control group [113]. Pembrolizumab is now approved by the USA (FDA) and European (EMA) regulatory bodies for use in patients whose tumors are MSI-H/dMMR regardless of histology and origin, making this drug the first tumor-agnostic molecularly targeted therapy to receive approval.

### 3.2. dMMR/MSI-High in PCa

MSI-high is not very frequently encountered in PCa arising in the general population, and MSI is not one of the leading pathways that drives prostate carcinogenesis [19]. Most of the cases harbor somatic mutations and ~20% of them are related to Lynch syndrome, mostly cases that are diagnosed before the age of 60 [112]. Sporadic MSI-high prostate cancers arise mostly as a consequence of deactivating mutations in MSH2 and MSH6, in contrast to colon and endometrial cancer, where the MSI-high status occurs through MLH1 epigenetic silencing. It is assumed that activated AR plays an important role in this process, through the promotion of DNA double-strand breaks [18,19,112,114,115]. 

MMR deficiency is seen in 5% of metastatic prostate cancer cases and is even less common in locally confined disease [15,16,17,18,116], with almost half of the MSI-high tumors presenting with metastatic disease [115,116]. Comparisons of primary hormone-naïve and their respective castrate-resistant metastatic tumors have shown that dMMR can be focal in the primary disease [116], indicating that dMMR in the advanced setting may develop through clonal selection. Mutations in the MSH2 gene have been shown as the most prevalent in one study [104], although the loss of MSH6 was most frequent in another study [117]. 

Histologically, MSI-high has been found in both adenocarcinomas and pure small cell carcinomas [104] and is usually associated with aggressive disease, high-grade pathology and the development of metastases [104,112,116]. Dense CD8+ lymphocytic infiltration and a higher mutational load have been associated with MSH2 loss [118]. An association of MSI-high with the presence of intraductal carcinoma and simultaneous *TP53* alterations has also been shown [115]. 

Clinically, a good response to ADT [112,115] and moderate sensitivity to docetaxel [115] has been observed in MSI-high tumors compared to MMR-proficient ones. More importantly, patients with dMMR or MSI-high prostate cancer show significant responses to the PD-1 inhibitor pembrolizumab. Initial approval for the use of pembrolizumab in MSI-high PCa was given in 2017 but was not specific to PCa. It was based on the results of five clinical studies (KEYNOTE-016, KEYNOTE-164, KEYNOTE-012, KEYNOTE-028, KEYNOTE-158) on colorectal and non-colorectal tumors. Among the non-colorectal patients, two patients had PCa and both showed some response. Since then, studies specifically for PCa patients have been reported, confirming the favorable effect of pembrolizumab in dMMR/MSI-high CRPC [104,119,120]. Based on these data, the use of pembrolizumab is suggested as a second and beyond line of treatment for patients with metastatic dMMR/MSI-high CRPC [121]. 

### 3.3. Biomarkers That Detect MMR Pathway Aberrations

Taking into consideration the worse prognosis of patients with dMMR/MSI-high tumors and the potential benefit of immunotherapy in this group of patients, guidelines recommend tumor testing for dMMR/MSI-high in patients with metastatic CRPC. Testing may also be considered in patients with regional metastases and patients with castration-sensitive metastatic PCa [112,122]. If dMMR or MSI-high is found, genetic counseling for Lynch syndrome is recommended. 

Tumor mutational burden (TMB) testing may also be considered in patients with metastatic CRPC [121]. A high TMB is defined as the accumulation of more than 10 mutations per megabase (Mb) [103,123] and corresponds to the presence of a variety of neoantigens that are recognized as foreign by the immune system, triggering immune responses [19]. PCa generally harbors 1–2 mutations per Mb, which is considered relatively low [124]. Patients harboring DNA damage repair mutations and HRD gather genetic alterations, due to the defective mechanisms of DNA repair, thus exhibiting a high mutational burden [71,124]. Nevertheless, the TMB does not always directly correlate with MSI status, meaning that MSI tumors can carry a high mutational burden, but not all tumors with an increased mutational load are MSI-high [125]. 

It is estimated that 2–3% of metastatic CRPC cases have a high TMB and simultaneous MMR deficiency [104]. Histologically, tumors with a high TMB often harbor ductal or intraductal histology and advanced Gleason grade (GG) (GG5) [124]. This population has extended survival when treated with nivolumab/ipilimumab, according to the CheckMate 650 study [71]. A recent study by Palmeri and associates confirmed the increased efficacy of immunotherapy in TMB-high or MSI-high neoplasms [125].

In 2020, the FDA approved pembrolizumab for the treatment of adult and pediatric patients with unresectable or metastatic tumor mutational burden-high (TMB-H) (≥10 mutations/megabase (mut/Mb)) solid tumors, based on the results of the KEYNOTE-158 trial [126], which included patients with tumors of various origins, including six patients with PCa, making TMB the second tumor-agnostic biomarker to receive approval. Based on these results, it is suggested that a high TMB can be used as an indication for the administration of pembrolizumab in patients with unresectable or metastatic PCa progressing after prior treatment (docetaxel and/or novel hormone therapy) and with no alternative treatment options [121,123]. However, its predictive value is still debatable, as the KEYNOTE-158 trial showed no remarkable survival benefit in TMB-high patients, although they responded well to immunotherapy [125]. Additionally, the cut-off point of 10 mut/Mb may be well documented for specific types of cancer, such as non-small-cell lung cancer, but there is evidence that when a higher threshold is used, such as 13 mut/Mb, the specificity of TMB as a biomarker is increased, although the sensitivity is decreased [125]. These ambiguous data underline the practical difficulties associated with biomarkers. 

dMMR/MSI-high and TMB are not 100% sensitive and specific, as not all patients with this tumor phenotype will respond to immunotherapy and, accordingly, immunotherapy responses have been noted in patients with tumors lacking these biomarkers. For example, monotherapy with anti-PD-1 (pembrolizumab) demonstrated an encouraging response in patients with metastatic CRPC, previously treated with docetaxel and anti-androgen therapy, with better results in bone-predominant or RECIST (Response Evaluation Criteria in Solid Tumours)-measurable CRPC [127]. In the same trial, a few cases that exhibited a durable response to anti PD-1 were microsatellite-stable, questioning the usefulness of MMR testing as an accurate predictive biomarker [86,127]. In another retrospective study of the effectiveness of pembrolizumab in heavily treated CRPC patients, >50% PSA declines were noted in patients whose tumors were MMS- and TMB-low [120]. Thus, there is an urgent need to identify additional biomarkers to guide therapeutic strategies. 

PDL-1 expression is being extensively used in a variety of neoplasms to inform therapeutic decisions regarding the use of PD/PD-L1 inhibition, the most prevalent example being non-small-cell lung carcinoma (NSCLC). In the KEYNOTE-001 study, NSCLC patients whose tumors expressed PD-L1, as assessed by immunohistochemistry, in >50% of the tumor cells had better response rates to pembrolizumab than patients with lower PD-L1 expression levels [128]. Thus, pembrolizumab was granted approval for use in NSCLC patients with >50% PD-L1 expression, representing the first time that a drug received approval simultaneously with its companion diagnostic test. 

There are a few immunohistochemical studies that have tested the expression of PD-1 and PD-L1 in prostate cancer [129,130,131], and the majority of them have shown increased expression in both the neoplastic tissue and the tumor-infiltrating lymphocytes compared to normal controls, with a gradual increase in expression with the progression of the disease stage [129] and in metastatic CRPC compared with the primary tumor [112]. A recent systemic meta-analysis of five case studies showed that PD-L1 was heterogeneously expressed in PCa and was correlated with a Gleason score greater than 7 and early biochemical recurrence after radical prostatectomy [132]. A relationship between PD-L1 expression in the lymphocytic infiltrate [131] or the tumor cells [133] and a worse prognosis has also been shown. 

An interplay between PD-L1 expression and the AR pathway has also been recognized. PD-L1 expression in the tumor cells has been correlated with AR levels [132,133,134] and the tumor proliferation index (assessed by Ki67 staining) [133], and PD-1 promoter methylation has been associated with AR activity [134]. PCa with aggressive features, such as a Gleason score >9, young age, advanced pathology staging and PSA level >10 ngr/mL, and increased PD-L1 expression in peritumoral lymphocytes was associated with lower survival rates [131]. Furthermore, it has been suggested that the PD-L1/PD-1 pathway is activated after hormonal inhibition and could represent a mechanism that contributes to enzalutamide resistance [124,132]. Regarding the role of PD-L1 expression as a predictive biomarker, the KEYNOTE-199 clinical trial showed that the effect of anti-PD-1 (pembrolizumab) monotherapy in metastatic CRPC was independent of PD-L1 expression in the tumor [86,135]. Another study, however, has shown that PDL-1 expression in ≥1% of the neoplastic cells is adequate to predict which patients will show a therapeutic benefit [71]. Given the complexity of the immune system, it is difficult to accurately predict patients’ responses based on only one biomarker [123]. 

### 3.4. Detection Methods of MSI Status

The MSI status can be detected by analyzing the lengths of various microsatellite markers by PCR (MSI-PCR), using commercially available kits or next-generation sequencing (NGS), and comparing them to that of normal tissue. For MSI-PCR, five microsatellite markers, known as the Bethesda panel—*BAT25*, *BAT26*, *D2S123*, *D5S346* and *D17S250* [136]—were initially recommended. However, mononucleotide repeats are more sensitive to transcription errors [137], and a panel of five mononucleotide repeats (BAT-25, BAT-26, NR-21, NR-24 and NR-22/NR-27) may be better suited to the identification of the MSI status of a tumor [138,139]. Based on the results of the comparison of the lengths of the five markers between normal and tumor tissue, neoplasms can be classified into three categories: MSI-high (MSI-H), indicating a difference in the length of ≥2 of the five markers; MSI-low (MSI-L), when only one marker exhibits a difference in its length; and microsatellite-stable (MSS), when all markers have the same length in the tumor and the non-neoplastic tissue [105,140,141]. Regarding NGS, microsatellite regions that coincidentally intervene in the sequence of a predefined gene panel are examined and compared to MSI-stable reference samples [142]. Tumors are generally considered unstable when they have ≥20% unstable loci [143], but the cut-off values vary depending on the guidelines that are being followed [142].

**Figure 3:**
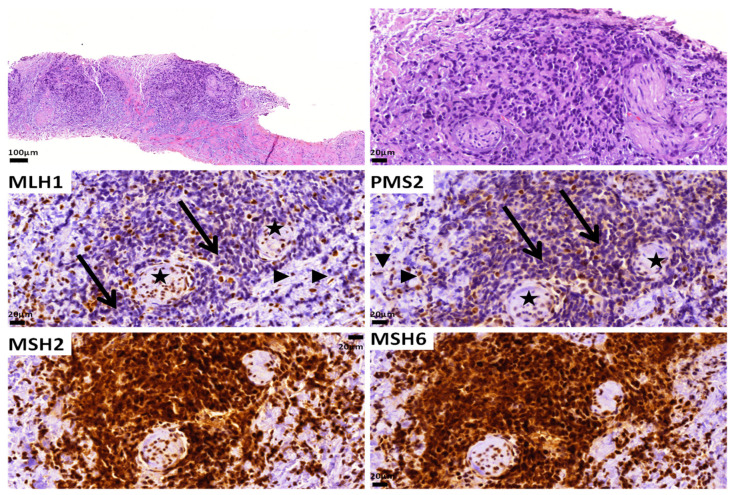
A Gleason score 9 (5 + 4) adenocarcinoma with dMMR as assessed by immunohistochemistry. Note the loss of MLH1 and PMS2 expression in the tumor cells (arrows). Positive stromal cells (arrowheads) and nerves (stars) serve as internal controls. MSH2 and MSH6 expression is retained. The patient did not have Lynch syndrome.

The MMR status can be detected by assessing the expression of the relevant proteins by immunohistochemistry [95]. When neoplastic cells retain the expression of all the proteins, then the tumor is considered MMR-proficient (pMMR), and the enzymes of mismatch repair are considered functionable. dMMR is defined by the loss of expression of one or more of the four MMR proteins (MLH1, MSH2, MSH6 and PMS2) [96]. As these proteins function as heterodimers, PMS2 and MSH6 are usually unstable without their respective partners, i.e., MLH1 and MSH2. Thus, when MLH1 is lost (due to mutation or hypermethylation of the MLH1 gene), PMS2 (its dimer partner) is also not expressed in the tissue. Similarly, when MSH2 is lost (due to mutation of its gene), MSH6 expression is also lost (Figure 3). In contrast, MLH1 and MSH2 are stable even when PMS2 and MSH6 are absent. Therefore, in the case of the loss of PMS2 or MSH6 (due to mutations of their respective genes), the expression of their partners, MLH1 and MSH2, respectively, is retained in the tissue [141,144]. 

MSI-PCR and immunohistochemistry (IHC) display a high level of concordance and they complement each other in regard to recognizing tumors with dMMR/MSI-high [141,145,146]. Advantages of IHC include its low cost, widespread availability, easy interpretation and low requirements in terms of tissue quantities [147]. It is the preferred method in cases with low tumor content (i.e., intense inflammation), as both PCR and NGS may not be sensitive enough to analyze the tumor cells’ DNA in such cases. In addition, the specific MMR gene that is lost and, thus, mutated can be identified with IHC, providing guidance for further genetic analyses for Lynch syndrome diagnosis. However, in up to 10% of cases, immunohistochemistry may be falsely positive, as a truncating mutation in the MMR gene, although rendering the gene inactive and the cells dMMR, does not affect the protein’s expression (expression is retained in IHC) [142,148]. In this case, IHC will be falsely positive for all MMR enzymes and a pMMR result will be given for the tumor. MSI-PCR will be able to correctly categorize the tumor as MSI-high in this setting. 

NGS has comparable results to MSI-PCR and immunohistochemistry [149], and the simultaneous analysis of multiple genetic aberrations [150,151], the estimation of the tumor mutational burden (see below) [71,123] and the application of a standardized and semi-automatized interpretation method reduces the mistakes arising from the human factor and shortens the time to diagnosis [142]. In addition, in some instances, paired normal tissue is not required [152]. In PCa, there are studies that show a discrepancy between MSI-PCR and MSI-NGS, with MSI-PCR having more presumably false-positive results [116], thus highlighting the need for more complex techniques for this malignancy.

Following the general trend of medicine towards less invasive methods, a promising, though not yet perfected, technique is the assessment of the MSI status on liquid biopsy specimens, i.e., those obtained from a peripheral blood sample, by applying commercially available NGS platforms to circulating tumor DNA [153]. These methods are based on massively parallel sequencing (MPS) and produce plenty of genomic data, although they are not yet fully functionable or largely available and are highly influenced by the percentage of circulating neoplastic cells; thus, their relevance increases in advanced disease [153]. Currently, there are a few commercially available pan-cancer MPS kits that analyze peripheral blood with enhanced specificity and sensitivity, providing information on a significant number genes and homopolymer regions, as well as the TMB and MSI status [153]. Future studies are expected to confirm their utility in PCa. 

### 3.5. Limitations in the Detection Methods of Biomarkers

It should be emphasized that several practical limitations that determine which detection method is better suited for each sample appear in daily practice. Apart from the specialized laboratory equipment that may be required in some techniques, there are also tissue limitations that guide the selection and application of each test. For instance, NGS’ challenges include not only the high cost and increased technical and bioinformatics-related demands but also a minimum DNA input prerequisite in order to accurately detect the presence of genetic alterations [154,155]. The DNA quantity requirement depends on the commercial platform used [156]. Available data suggest that 6.25 ng DNA can be sufficient for specific commercially available kits, but only when this amount of DNA is derived from intact genomic DNA and not when derived from formalin-fixed paraffin-embedded (FFPE) tissue [156]. The DNA requirements of NGS in FFPE tissue, which are the most widely encountered specimens in routine practice, can be significantly higher—for example, >100 ng in the Illumina platform [157]. Additionally, a minimum tumor content of >10% tumor cells is a prerequisite for the recognition of copy number variations [157]. Finally, analysis should be performed in both exons and non-coding DNA regions, as a significant proportion of genomic alterations take place in introns [112], increasing the cost and the complexity of the analysis.

On the contrary, PCR-based analysis is more affordable than NGS and requires smaller amounts of DNA, i.e., DNA extracted from five 5-μm-thick sections, with at least 10% tumor cell content [158]. Nonetheless, it is not yet widely available and is more costly, compared to IHC, as it requires specialized equipment and expertise that are not readily available in all settings. In addition, it cannot point to the MMR protein that is at fault and, consequently, cannot guide further genetic testing. Its accuracy is dependent on the tumor cell content, and cases with <10% tumor cells are generally not suitable for PCR analysis, unless tumor enrichment can be performed. Finally, technical issues and previous therapy may affect all techniques, and preanalytical factors should be thoroughly taken into account [147]. 

The accuracy of the PCR method is dependent on the microsatellite markers that are analyzed. Most of the literature has been focused on colorectal carcinoma, as this tumor type is most commonly associated with an MSI-high status. Unfortunately, there is evidence that other tumors may be characterized by alterations in other microsatellite markers [159] not included in the commercially available panels. However, as technology improves and NGS becomes readily available, more widespread use may be anticipated, as NGS with the analysis of multiple loci may prove more sensitive in detecting an MSI-high status across tumor types. 

Finally, all these methods are considered complementary and, when indicated, the results of the molecular techniques should be correlated with the immunohistochemical results [96,99,142]. It should be mentioned that the majority of these methods have been evaluated in non-prostate malignancies, namely colon and endometrial cancer, and were only recently validated in studies that focused on PCa [112,142]. Guidelines suggest using an NGS assay specifically validated for prostate cancer [121], as some of the widely used techniques, such as MSI-PCR, have reduced sensitivity for PCa [142]. Testing for dMMR by performing IHC for the four proteins is also recommended. 

The relationship between MSI status and PD-L1 expression is not fully clarified. There are a few publications that correlate increased PD-L1 expression with the loss of more than two MMR enzymes [117,123]. It is believed that MSI-high tumors start to overexpress various immune-related genes and, among them, PDL-L1 is also enhanced [134]. In general, there is a varying percentage, ranging from 1 to 12%, of metastatic CRPC that is simultaneously MSI-high and PD-L1-high, while the frequency of concurrent PD-L1 overexpression and altered *BRCA* is not well described [123]. 

### 3.6. Predictive Biomarkers under Investigation

Experimental models have shown that SPOP mutations are associated with decreased ubiquitination and the enhanced expression of PD-L1. Thus, patients whose tumors harbor *SPOP* mutations, the second most common genetic alteration and the most common mutation in PCa, have a higher likelihood of responding to anti-PD-L1 therapy [103]. The loss of PTEN, which is also common in PCa, seems to diminish immune responses to neoplastic cells through the activation of the Interferon-1 pathway and the JAK2/STAT3 cascade [103]. It remains to be seen whether the identification of the molecular pathogenesis of the neoplasm may help to inform the selection of therapy. 

In other malignancies, tumor-infiltrating lymphocytes (TIL) have been used, along with the TMB and MMR status, as predictive factors for ICI [160]. Nonetheless, the data so far regarding TILs in PCa are quite limited [160]. Other biomarkers that have been identified as potentially predictive of the response to pembrolizumab in patients with PCa include the presence of mutations in the low-density lipoprotein receptor-related protein 1b gene (*LRP1b*) (75% response rate in LRP1b-mutated tumors vs. 14% in tumors without LRP1b mutations in a retrospectively reviewed cohort of 48 men who received ≥1 cycle of pembrolizumab for mCRPC, with high correlation between LRP1b mutations and TMB-high) [120]. As the high prevalence of LRP1b mutation has been shown in PCa [161] and a correlation between LRP1b mutation and the ICI response has been shown in melanoma patients [162], the authors hypothesized that LRP1b mutations may represent a surrogate marker for the mutational load [120].

Another biomarker potentially predictive of microsatellite instability is exostosin-like glycosyltransferase 3 (EXTL3). Data from The Cancer Genome Atlas (TCGA) highlight a potential interplay between MSI-high status and the expression levels of EXTL3 [163]. In addition, the expression of this molecule has been associated with rich peritumoral immune cell infiltration in various malignancies, suggesting a relation between EXTL3 and immune regulation [163]. Neoplasms with high EXTL3 expression were more likely to respond better to immunotherapy, and specifically to the anti-PD-L1 drug atezolizumab [163]. Interestingly, in other databases, such as CellMiner, the expression of this molecule was correlated with multiple drug responses, including Palbociclib and Rapamycin [163].

A contemporary analysis, using cBioportal data from various neoplasms, revealed an association of aberrations in the PARP1 gene with an elevated TMB and better overall survival rates, when this subgroup of patients was treated with ICI inhibitors [164]. Furthermore, CDK12 is involved in HRD [45] and has been found to be mutated in a small percentage of metastatic CRPC [66,67]. CDK12 mutations are used as an indication for the administration of the PARPi Olaparib as a second-line treatment for mCRPC. However, when CDK12 is mutated, it creates neoantigens due to the enhancement of gene fusion formation, and this may explain the observed response to PD-1 inhibitors [50,81,82,87]. Further studies are needed to strengthen the role of CDK2 as a predictive biomarker of the ICI response.

## 4. Future Therapeutic Perspectives and Emerging Biomarkers

Future perspectives in therapeutic interventions include novel pharmacological combinations [23,26]. The combination of anti-CTLA4 and anti-PD-1 therapy in the CheckMate 650 clinical trial yielded satisfactory results in both groups of patients that were enrolled (before and after cytotoxic chemotherapy), but it was accompanied by significant toxicity; thus, dosage adjustment was suggested [135]. Exploratory analyses identified potential biomarkers predictive of a therapeutic benefit that were similar to those described above, such as the TMB, mutations in genes involved in DNA damage repair and HRD, PDL-1 expression, MMR status and mutations in *CDK12* [135]. Pembrolizumab has been combined with Olaparib in docetaxel-resistant metastatic CRPC carrying *BRCA* or *ATM* mutations with a beneficial response, although the molecular background of the tumor was not clear and the method of PD-L1 expression assessment was not mentioned in the study [123].

The combination of novel anti-PD-L1 antibodies such as atezolizumab with novel anti-CTLA4 agents such as cabozantinib is also being tested in phase 3 clinical trials (NCT04446117), in CRPC patients previously treated with abiraterone or enzalutamide [78,165]. Clinical trials that test the efficacy of pembrolizumab with enzalutamide are recruiting patients with advanced hormonal-sensitive PCa (KEYNOTE-991: NCT04191096) and metastatic CRPC (KEYNOTE-641: NCT03834493) [26]. In both trials, tissue and blood samples are obtained from the participants for further analysis, whereas the KEYNOTE-991 trial also semi-quantifies PDL-1 expression in patients’ tumors with IHC [166,167]. The therapeutic results along with emerging predictive tissue biomarkers are anticipated to be published soon. A summary of the up-to-date approved DDR targeting drugs/predictive biomarkers is shown in Table 1.

Finally, indirect markers of oxidative DNA stress targeting less specific DNA repair pathways, such as 8-Hydroxy-2-Deoxyguanosine (8-OHdG) and 8-Iso-Prostaglandin F2α (8-iso-PGF2a), have been introduced recently in PCa [168]. They represent end-products of guanine oxidation and indicate the activation of cyclooxygenase-mediated inflammation [168]. Oxidative stress correlates with DNA damage and is implicated in carcinogenesis in various malignances [169]. There is also evidence that malondialdehyde (MDA), which is produced by the peroxidation of polyunsaturated fatty acids, is increased in PCa compared to healthy individuals and benign hyperplasia [170]. These molecules can be detected in urine with liquid chromatography–tandem mass spectrometry, and thus their levels can be monitored without any surgical intervention [171]. Recently, 8-OHdG and 8-iso-PGF2a were studied in PCa as predictive biomarkers of the completeness or radicality of prostatectomy in patients who underwent robot-assisted radical prostatectomy [171]. Di Minno and associates proposed that the levels of these two oxidation products within normal limits three months postoperatively reflect the complete removal of the tumor and presumably a lower recurrence rate [171]. However, further studies with larger sample sizes are required before definitive conclusions can be drawn; these studies should incorporate longer follow-up periods, whereas the lack of tissue specificity should also be taken into account.

## 5. Conclusions

In conclusion, DNA damage repair mechanisms represent a promising but not fully explored pathway in prostate pathogenesis. Targeted therapies provide a survival benefit with tolerable toxicity in a highly selective subset of patients. HRD mutations and dMMR/MSI-high status represent biomarkers used to identify the population most likely to benefit from these personalized interventions. Currently, albeit slightly variable across regions, most national and international guidelines suggest somatic tumor testing for alterations in BRCA1, BRCA2, ATM, PALB2, FANCA, RAD51D, CHEK2 and CDK12 (homologous recombination genes) in patients with metastatic prostate cancer and for MSI-H/dMMR in patients with metastatic CRPC. The latter can be considered in all patients with metastatic disease. Both sets of biomarkers can also be considered in patients with regional disease. The results of these tests will inform therapeutic decisions, i.e., the use of PARPi and anti-PD1 therapy, respectively, thus providing a basis for precision oncology. However, there is still a long way to go until predictive biomarkers acquire high sensitivity and specificity. Newer biomarkers, and, most importantly, combinations of them, as was shown in the impressive AstroPath platform in melanoma patients [172], hold promise for more efficient use. Future research may elucidate the timeline during the progression of PCa wherein these mechanisms become prominent in carcinogenesis, as well as assisting the development of reproducible and reliable biomarkers. 

## Figures and Tables

**Table 1:** Summary of the currently FDA-approved drugs/biomarkers targeting DNA repair mechanisms.

Drug	Approved Indication	Biomarker	Clinical Trial
Olaparib	Second-line therapy after failure of treatment with second-generation antiandrogen agents or docetaxel or third-line treatment	*BRCA1*, *BRCA2*, *ATM*, *BRIP1*, *BARD1*, *CDK12*, *CHEK1*, *CHEK2*, *FANCL*, *PALB2*, *PPP2R2A*, *RAD51B*, *RAD51C*, *RAD51D* or *RAD54L* mutations	NCT02987543
Rucaparib	Second-line therapy after failure of treatment with second-generation antiandrogen agents or docetaxel or third-line treatment	*BRCA1*, *BRCA2* somatic and germline mutations	NCT02952534 NCT02975934
Pembrolizumab	Unresectable or metastatic PCa progressing after prior treatment (docetaxel and/or novel hormone therapy) and with no alternative treatment options	MSI-high/dMMR, tumor mutational burden ≥10 mutations/megabase	KEYNOTE-158/ NCT02628067
**Ongoing trials with biomarkers**			
Niraparib	Metastatic CRPC	Inclusion biomarker criteria: (a) biallelic DNA repair anomaly based on a sponsor-validated blood or tissue assay; (b) germline pathogenic *BRCA1* or *BRCA2* mutation by any test	NCT02854436 (phase 2)

## Data Availability

Not applicable.
